# Feasibility of performance-based and self-reported outcomes in self-managed falls prevention exercise interventions for independent older adults living in the community

**DOI:** 10.1186/s12877-022-02851-9

**Published:** 2022-02-22

**Authors:** Linda Mansson, Beatrice Pettersson, Erik Rosendahl, Dawn A. Skelton, Lillemor Lundin-Olsson, Marlene Sandlund

**Affiliations:** 1 Department of Community Medicine and Rehabilitation, Section of Physiotherapy, Umeå University, Umeå, Sweden; 2 School of Health and Life Sciences, Glasgow Caledonian University, Glasgow, UK

**Keywords:** Aged, Falls prevention, Patient outcome assessment, Self-managed

## Abstract

**Background:**

Little is known about associations between performance-based measurements and self-reported scales, nor about ceiling effects or sensitivity to change to evaluate effects in the target population for self-managed exercise interventions. This study aimed to explore the feasibility of using performance-based outcomes for gait speed, functional leg strength and balance, and self-reported outcomes of falls-efficacy and functional ability in two self-managed falls prevention exercise interventions for community dwelling older adults.

**Methods:**

Independent living, community-dwelling older adults (*n* = 67) exercised with one of two self-managed falls prevention exercise programmes, a digital programme (DP) or a paper booklet (PB) in a 4-month participant preference trial. Pre- and post-assessments, by blinded assessors, included Short Physical Performance Battery (SPPB) and 30s Chair stand test (30s CST). Participants completed self-reported questionnaires: Activities-specific and Balance Confidence scale (ABC), Iconographical Falls Efficacy Scale (Icon-FES), Late-Life Function and Disability Instrument Function Component (LLFDI-FC). In addition, improvement in balance and leg strength was also self-rated at post-assessment. Participants’ mean age was 76 ± 4 years and 72% were women.

**Results:**

Ceiling effects were evident for the balance sub-component of the SPPB, and also indicated for ABC and Icon-FES in this high functioning population. In SPPB, gait speed, 30s CST, and LLFDI-FC, 21–56% of participants did not change their scores beyond the Minimal Clinically Important Difference (MCID). At pre-assessment all performance-based tests correlated significantly with the self-reported scales, however, no such significant correlations were seen with change-scores. Improvement of performance-based functional leg strength with substantial effect sizes and significant correlations with self-reported exercise time was shown. There were no differences in outcomes between the exercise programmes except that DP users reported improved change of leg strength to a higher degree than PB users.

**Conclusion:**

The LLFDI-FC and sit-to-stand tests were feasible and sensitive to change in this specific population. The balance sub-component of SPPB and self-reported measures ABC and Icon-FES indicated ceiling effects and might not be suitable as outcome measures for use in a high functioning older population. Development and evaluation of new outcome measures are needed for self-managed fall-preventive interventions with high functioning community-dwelling older adults.

**Supplementary Information:**

The online version contains supplementary material available at 10.1186/s12877-022-02851-9.

## Background

Falls prevention is important as the ageing population increases, and the occurrence of falls rise [1]. Exercise based falls prevention programmes can significantly reduce the risk of falls and rate of falls in community-dwelling older adults [2], and may also reduce fall related psychological concerns like fear of falling [3]. The fall preventive effect is dependent on the amount of exercise time, or dose [4]. Unfortunately, previous studies for home-based falls prevention have shown generally poor adherence [5], and engagement is known to decrease over time [6]. One suggestion to increase adherence has been to support older adults’ active involvement in their own exercise routines [7]. Through self-management older adults can be supported to take responsibility for their falls prevention, which could impact their daily lives and also reduce the need for health care services. Traditionally self-managed exercise has been introduced with demonstration and verbal instructions, reinforced by written text and illustrations. Today, however, the support for self-management can also be provided by digital technology. Digital technology seems to provide more support in self-management of exercise than a paper booklet [8] and can also support adherence, provide feedback, facilitate documentation and registration of adherence. In the future, smartphone technology may also enable self-assessed outcome measurements [9–11] to motivate self-management of exercise and falls, and objectively monitor change in function.

Outcome measures that are sensitive enough to detect important differences in physical capacity are crucial to evaluate the effects of exercise interventions. Older adults are a heterogenous group and it is therefore important to evaluate psychometric properties for the intended target group. For high functioning community-dwelling older adults, commonly used outcome measures might have ceiling effects, which can limit evaluations of exercise effects [12–14]. When it comes to self-managed exercise interventions, it would be preferable to use self-reported outcomes as they can be used when occasions for performance-based measures are limited. Previous studies have shown performance-based and self-reported measures to be comparable for physical function, for example in measurements after hip fracture [15]. Further investigation of these relationships, between performance-based measurements and self-reported outcomes, may increase our understanding and ability to interpret the results from outcomes used in a self-managed intervention.

The overall aim of this study was to explore the feasibility of using performance-based outcomes of gait speed, functional leg strength and balance, and self-reported outcomes of falls-efficacy and functional ability in two self-managed falls prevention exercise interventions. More specifically to investigate the feasibility of the outcome measures regarding ceiling effects and sensitivity to change, and to explore associations between performance-based measurements and self-reported scales. We also explored measured effects and differences depending on format of the programme (digital vs. paper).

## Methods

The intervention was a participant preference trial in which the participants choose to self-manage their exercise with either a digital programme (DP) or a paper booklet (PB). Each participant started the intervention by attending an introduction meeting, including individual pre-assessments and a short presentation of the exercise programme of their choice. The intervention included four months of fully self-managed exercise, guided by the chosen programme, and the intervention ended with a post-assessment meeting. The study complies with the Declaration of Helsinki and received approval from The Regional Ethical Review Board in Umeå (Dnr 2016/106–31). All participants were given written and verbal information and gave written informed consent. The study was registered on ClinTrials.gov 28/09/2016, with ID: NCT02916849.

### Participants

Recruitment was performed in senior citizen organizations and at one health care centre. At the senior organizations the participants were recruited by members of the research team and at the health care centre by health care professionals. Inclusion criteria were: ≥70 years old, living independently in the community, able to rise from a chair and stand without support, experiences of deterioration in balance and/or need to be more careful not to lose balance and/or have experienced a fall the past year. Exclusion criteria were: doing physical exercise more than three hours per week, self-reported progressive disease that was likely to influence mobility and cognitive difficulties. Status of cognitive condition was judged during the screening interview, if the person was able to answer questions satisfactorily and able to converse about matters regarding the study, they were considered suitable to take part in the study. The study participants were 72% women and the mean age was 76 years.

### Intervention

Both programmes were fully self-managed, meaning the programme was used without professional guidance and it could be tailored and progressed by the participant as often as desired. The PB was based on the Otago Exercise Programme [16], the DP exercises were similar but expanded with both easier and more challenging exercises inspired by the Falls Management Exercise Programme (FaME) [17]. The digital programme (Safe Step v1 web-based or mobile application) was accessed by computer, smartphone or tablet [8, 18, 19]. The exercises were presented in short video clips with verbal instructions and organized into ten predetermined groups of balance, strength, and step/gait exercises, all including several exercise alternatives of various difficulty to choose from. In the PB the exercises were organized into strength or balance exercises, each with three different levels of difficulty. All participants were instructed to choose a set of ten exercises that they felt would be challenging but not too demanding. Balance exercises should give sensation of unsteadiness but without losing the balance with risk of a fall. For strength exercises, an exertion should be noticed but still be able to complete the stipulated number of repetitions. Participants were recommended to exercise for at least 30 min three days per week, and to adjust the programme by selecting new exercises whenever the current ones became too easy or too difficult to perform.

All participants attended an introduction meeting for approximately two hours including a short lecture on falls and falls prevention followed by physical assessments, completion of self-administered questionnaires, and an introduction of either the DP or PB. As self-management was the focus of the intervention, interactions with participants were limited during the study. A contact number was provided in case of any emerging technical problems during the intervention and all participants received a telephone call a few weeks after study start to identify any potential problems with the programmes. For some of the participants in the DP group more interaction was scheduled, six participants had an observation in their own home midway in the intervention, and twelve participants were in a sub-group who were offered monthly peer-mentor meetings. Further detailed description of the interventions can be found in the Supplementary Table (S1), Template for Intervention Description and Replication (TIDieR) checklist. In a previously published paper on adherence [19] more details about this feasibility study is reported.

### Data collection and outcome measures

At the introduction meeting, sociodemographic characteristics, access to technology, self-reported health, function, and mobility, as well as history of falls in the past year, was collected through a self-report questionnaire. One of the researchers was present in the room to answer questions when participants filled in the questionnaires. A physiotherapist blinded to group allocation, and previous results, performed all measurements of physical functioning at the pre- and post-assessment.

#### Performance-based outcome measurements

Functional performance was measured using the Short Physical Performance Battery (SPPB) [20]. The SPPB is a valid, reliable and responsive instrument [21] that comprises standing balance, gait speed over four meters, and Five times sit-to-stand test. A total score of 0–12 is formed from the sub-components, higher score indicate a better functional performance. Standing balance was assessed in three positions: feet together, semi tandem, and full tandem. Gait speed was timed at preferred gait speed over four meters. The test was repeated twice, and the fastest time was registered as test result. Preferred walking speed is a reliable and valid measurements to use for older adults [22]. The 4-m gait speed has been found to predict disability almost as well as the total SPPB [23]. The Five times sit-to-stand (5TSTS) is a timed performance of the participants’ ability to sit and stand five times as quickly as possible from a chair with arms folded over the chest. The 5TSTS can be used to assess functional leg strength [24], and identify older adults with impaired balance [25] and fall risk [26]. The test showed good test-retest reliability ICC 0.89; 95% CI = 0.79–0.95 [26].

The 30s Chair stand test (30s CST) was used to assess functional leg strength and endurance [27]. During the assessment, the participant’s ability to perform a maximum number of sit-to-stands with arms folded over the chest, in 30 s was counted. The number of correct stands was noted, if the last stand was more than halfway up it was also counted. The 30s CST has been found to have good test-retest reliability (intra-class coefficient *R* = 0.89; 95% CI = 0.79–0.93) and concurrent validity with 1 RM leg-press among community-dwelling older adults (*r* = 0.77, 95% CI = 0.64–0.85 [27].

#### Self-reported outcome measures

Different definitions and tests have been used to describe and report fear of falling, where loss of confidence in balance abilities to function safely may cause fear and avoidance of activities [28].

Self-reported balance confidence was measured using a Swedish version of the Activities-specific Balance Confidence Scale (ABC) showing internal consistency Cronbach’s α = 0,95–0,97, and test-retest ICC 0.82, 95% CI = 0.72–0.88 [29]. Verbal instructions were provided individually for this questionnaire. Confidence in performance of a task without losing balance or becoming unsteady is rated on a scale from 0 to 100% in 16 ambulatory activities [30], higher scores indicate more confidence. The ABC scale has been found both valid and reliable for assessing balance confidence in community-dwelling older adults [30], as well as excellent correlation with performance-based measures such as the Timed Up and Go Test (TUG) and the SPPB [31].

Fear of falling was measured by the 30-item Iconographical Falls Efficacy Scale (Icon-FES) a picture-based questionnaire [32], translated to Swedish for the purpose of this study. The translation procedure included some clarifications of items, i.e., three items concerning reaching for something above your head was clarified regarding if one stood on the floor, a stool, or a ladder when reaching. Additionally, some linguistic adaptations were made, e.g., “Walking around in the neighbourhood” was changed to “Take a short walk”. The process of translation followed established guidelines [33] and was approved by the developers. Fall related concern is rated on a 4-point scale, ranging from not at all concerned to very concerned, by looking at pictures including a short descriptive text of a variety of activities and situations. Higher scores reflect a greater fall related concern. The Icon-FES has excellent psychometric properties and has been recommended for use among high functioning older adults living in the community showing internal consistency Cronbach’s α = 0,96, and test-retest ICC 0.90, 95% CI = 0.83–0.94 [32].

The Late-Life Function and Disability Instrument (LLFDI) measures functional limitations and disability in community-dwelling older adults [34, 35]. In this study the Swedish translation was used [36] as a questionnaire [37], with the permission from the developers. Verbal instructions were provided individually. The function component (LLFDI-FC) is assessed through 32 different physical activities and comprise a general scale of function and three sub-scales: upper extremity, basic lower extremity and advanced lower extremity. The raw scores of the scale are transformed to a standardized score of 0–100, where a higher score indicates better levels of functioning. The LLFDI-FC is frequently used in research with community-dwelling older adults and has been shown to have high construct validity, high test-retest reliability and to be sensitive to change [38, 39]. The Swedish cross-cultural translation showed internal consistency Cronbach’s α = 0,96, and test-retest ICC 0.91 [36]. The function component has also been shown to predict falls and functional decline in older community-dwelling adults with mobility limitations [39].

Self-rated improvements in leg strength and balance were assessed by two single-item questions with a 5-point Likert scale (“My leg strength/balance has improved by training with the program”, 1 = disagree strongly and 5 = agree strongly). The questions were developed by the research group for the purpose of this study.

### Data analysis

Participant characteristics are reported using means with standard deviations or as proportions when applicable. In this feasibility study both groups received an exercise intervention (DP and PB). To improve power in analyses of the outcome measures data were pooled from both groups. Differences in effects between groups were explored although the study was not powered for this purpose. Pre-post values are summarized using non-parametric measures of central tendency and dispersion (median and interquartile range). Eventual occurrence of ceiling effects were explored by examining pre-assessment median values and the scores for the third or first quartile depending on whether a high or low score was considered superior. For outcomes with no ceiling effects, and available limit values for Minimal Clinically Important Difference (MCID), the proportions of participants who improved or deteriorated were calculated. For SPPB the MCID applied was 1 point [40], for gait speed 0,1 m/s [41], for 30s CST 2 repetitions [42] and for LLFDI-FC 2 standard scores [43]. Changes after the intervention for the whole group were analysed using Wilcoxon signed-rank test and effect sizes calculated as Rank biserial correlations meaning the difference between the proportion with improved scores and the proportion with decreased scores [44]. For comparisons of changes between groups (DP or PB) the Mann-Whitney U-test was used. To explore associations between effects and exercise time and associations between performance-based and self-reported outcomes, Spearman’s rank correlations were calculated. Results were interpreted according to the following levels; very high correlation (0.90 to 1.00), high correlation (0.70 to 0.90), moderate correlation (0.50 to 0.70), low correlation (0.30 to 0.50) or poor (less than 0.30) [45]. Alpha was set to 0.05. All analyses were performed in the software Jamovi version 1.6.3. Retrieved from https://www.jamovi.org, The jamovi project (2020).

## Results

In total, 67 participants were enrolled in the study (Table 1). Over 90% experienced that their balance had been reduced, and 58% reported at least one fall the past year. Nearly 60% reported having a good or very good health, however among men only 37% reported good or very good health. In the whole group 60% had access to a smartphone/tablet and 82% to a computer, more women had a smartphone/tablet and more men a computer. Regarding choice of programme 43% preferred to exercise with the DP and the rest preferred the PB, almost 2/3 of participants who chose the DP were women and for the PB more than 3/4 were women. During the 16 weeks of exercise, five participants (17%) in the DP group and 14 (37%) in the PB group withdrew from the study, resulting in 48 participants completing the study. The only significant difference for pre-assessment variables for participants who withdrew was slower gait speed (*p* = 0.009). A few participants who withdrew still filled in self-reported scales at the end of the study. More information on attrition and adherence from this feasibility study has been reported elsewhere [19]. The mean exercise time per week for the whole sample was 53 min; 63 min for DP and 46 min for PB group. Two non-injurious falls during exercise were reported by one individual in the PB group. No other adverse events were reported.

**Table 1:** Characteristics of included participants, gender and programme preference

	Women *n* = 48	Men *n* = 19	Total *n* = 67	DP *n* = 29	PB *n* = 38
Age, years, mean ± SD	76 ± 4	76 ± 5	76 ± 4	76 ± 5	77 ± 3
Total exercise minutes, mean ± SD	745 ± 685	1116 ± 713	850 ± 708	1008 ± 810	730 ± 602
Women, n (%)			48 (72)	18 (62)	30 (79)
Living alone, n (%)	26 (54)	4 (21)	30 (45)	13 (45)	17 (45)
Education, n (%)					
Primary	28 (58)	6 (32)	34 (51)	11 (38)	23 (61)
Secondary	11 (23)	8 (42)	19 (28)	9 (31)	10 (26)
Tertiary	9 (19)	5 (26)	14 (21)	9 (31)	5 (13)
Perceive decreased balance, n (%)	44 (92)	18 (95)	62 (93)	26 (90)	36 (95)
Self-reported falls previous year	26 (54)	13 (68)	39 (58)	17 (59)	22 (58)
Use of walking aid, n (%)					
Indoors	2 (4)	0 (0)	2 (3)	0 (0)	2 (5)
Outdoors	12 (25)	2 (11)	14 (21)	4 (14)	10 (26)
Self-reported health, n (%)					
Good or very good	31 (65)	7 (37)	38 (57)	16 (55)	22 (58)
Fair	15 (31)	11 (58)	26 (39)	13 (45)	13 (34)
Not good	2 (4)	1 (5)	3 (4)	0 (0)	3 (8)
Access to smartphone/tablet, n (%)	30 (65)^a^	9 (47)	39 (60)	23 (79)	16 (44)^a^
Access to computer, n (%)	37 (49)^a^	16 (84)	53 (82)	26 (90)	27 (75)^a^

^a^ missing n = 2; *DP* Digital Programme, *PB* Paper Booklet

### Feasibility of the outcome measures

Median values, interquartile ranges and effect sizes are presented in Table 2. In the balance test, from the SPPB, 70% of the participants managed the highest possible score (level 4) indicating an evident ceiling effect. The falls-efficacy questionnaires ABC and Icon-FES also indicated ceiling effects. The median value for ABC total score was 84 out of a maximum of 100 points and 25% reached scores over 89.5 points. For Icon-FES the median score at pre-assessment was 47 and 25% scored below 40.5 points with 30 being the best score out of 120 possible. The SPPB total score was in the higher range with a median score of 9 and 25% scoring above 10 points of 12 at pre-assessment. No ceiling or floor effects were indicated for the LLFDI-FC. For gait speed and chair stands, ceiling effects are not obtainable as they are a timed measure.

**Table 2:** Absolute values for the whole sample and statistical differences between pre- and post-assessments. *P*-values for differences between the groups, digital programme (DP) and the paper booklet (PB)

	n Pre post	Median (Q1-Q3) Pre post	***p***-value^a^ within group	Effect size (***r***_**rb**_)^**b**^	DP Pre: n 29^c^ Post: n 24^c^	PB Pre: n 38^c^ Post: n 24^c^	***p***-value^d^ between groups
SPPB, (0–12)	67	9 (8–10)	0.058	0.34	10 (8–10)	9 (8–10)	0.522
	48	10 (9–11)			10 (9–11.3)	9.5 (7.75–11)	
Gait speed 4 m, m/s	67	0.8 (0.7–1.0)	0.204	0.21	0.9 (0.7–1.0)	0.8 (0.6–0.9)	0.976
	48	0.9 (0.7–0.9)			0.9 (0.8–0.9)	0.8 (0.6–0.9)	
Balance score (0–4)	67	4 (3–4)	0.842	−0.07	4 (3–4)	4 (3–4)	0.728
	48	4 (4–4)			4 (4–4)	4 (3–4)	
5TSTS, sec	64	15.4 (13.1–17.6)	**< .001**	−0.65	15.5 (13.9–16.9)^−1^	15.1 (13.0–19.7)^−2^	0.846
	48	14.1 (11.9–15.3)			14.5 (11.2–15.2)	13.6 (12.0–15.5)	
30s CST, n	65	11 (10–13)	**< .001**	0.76	11 (10–12.3)^−1^	11 (9–13)^− 1^	0.870
	47	12 (10–13)			12 (10–13.5)^−1^	12 (10–13)	
ABC, total score (0–100)	67	84.0 (73.0–89.5)	0.520	0.10	85.0 (73.0–92.0)	83.5 (69.5–89.0)	0.715
	53	86.0 (75.0–93.0)			87.0 (75.0–93.0)^+ 3^	83.0 (71.5–90.8)^+ 2^	
Icon-FES, total score (30–120)	67	47.0 (40.5–56.5)	0.391	−0.14	49.0 (38.0–55.0)	46.5 (42.3–58.8)	0.236
	53	46.0 (38.0–54.0)			43.0 (38.5–51.5)^+ 3^	47.0 (39.3–61.5)^+ 2^	
LLFDI-FC, scaled score (0–100)	66	62.7 (54.9–70.4)	0.420	0.13	63.0 (58.0–71.3)	62.5 (54.5–68.8)^−1^	0.298
	53	63.5 (56.9–71.3)			66.8 (59.6–71.3)^+ 3^	61.0 (54.7–68.5)^+ 2^	
Upper extremity	66	82.0 (71.0–88.0)	0.836	0.04	82.0 (71.0–82.0)	82.0 (68.4–88.0)^−1^	0.496
	53	82.0 (73.9–88.0)			82.0 (73.9–88.0)^+ 3^	82.0 (71.7–88.0)^+ 2^	
Basic lower extremity	66	69.4 (64.3–81.2)	0.172	0.23	72.1 (67.2–88.0)	68.6 (61.8–77.2)^−1^	0.557
	53	72.1 (64.8–81.2)			74.3 (70.2–84.6)^+ 3^	68.7 (62.1–81.2)^+ 2^	
Adv. lower extremity	66	57.0 (45.8–66.2)	0.893	0.02	57.0 (49.7–67.1)	55.9 (45.8–63.6)^−1^	0.290
	53	58.2 (45.8–69.1)			59.4 (51.3–69.1) ^+ 3^	54.8 (45.1–66.2)^+ 2^	
Self-rated balance improvement (1–5)	53	4 (3–4)			4 (3–5) ^+ 3^	4 (3–4) ^+ 2^	0.111
Self-rated leg strength improvement (1–5)	53	3 (3–4)			4 (3–4.5) ^+ 3^	3 (3–4) ^+ 2^	**0.033**

*SPPB* Short Physical Performance Battery, *5TSTS* Five times sit-to-stand, *30s CST* 30 s chair stand test, *ABC* Activities-Specific Balance Confidence Scale, *Icon-FES* Iconographical Falls Efficacy Scale, *LLFDI-FC* Late-Life Function and Disability Instrument-function component

^a^ *Wilcoxon* signed-rank test; ^b^ *r*_rb;_Rank biserial correlation; ^c^ numbers after the parentheses indicate missing or extra data (participants who withdraw could participate in questionnaire by mail). ^d^ *Mann*–*Whitney U*-test. The study was not powered for effects

In terms of sensitivity to change, about half of the participants improved according to limits of MCID (Table 3) for the SPPB [40] and 30s CST [42], while nearly a third deteriorated in gait speed [41]. For LLFDI-FC [43] about one third each improved, deteriorated or did not change. Between 21 and 56% of participants did not improve nor deteriorate between pre- and post-assessment according to established limits of MCID these four outcomes.

**Table 3:** Number and proportions of participants who improved or deteriorated according to limits of minimal clinically important difference (MCID)

	n	Improved n (%)	Deteriorated n (%)	No change n (%)
SPPB, MCID 1 point	48	27 (56)	11 (23)	10 (21)
Gait speed, MCID 0.1 m/s	48	7 (15)	14 (29)	27 (56)
30s CST, MCID 2 stands	47	21 (45)	2 (4)	24 (51)
LLFDI-FC, scaled score (0–100), MCID 2 points	53	18 (34)	14 (26)	21 (40)

SPPB, Short Physical Performance Battery; 30s CST, 30 s chair stand test; LLFDI-FC, Late-Life Function and Disability Instrument-function component

### Associations between performance-based measurements and self-reported scales

At pre-assessment, the performance-based tests SPPB, gait speed, balance, and 30s CST all had significant low to moderate correlations with self-reported scales, with the exception of poorer correlations between balance and Icon-FES (Table 4). For the 5TSTS significant poor correlations with all the self-reported scales ABC, Icon-FES, and LLFDI-FC was shown.

**Table 4:** Correlations between the performance-based measurement of gait speed, balance and functional leg strength with self-reported scales for falls-efficacy and functional ability at pre-assessment

	SPPB	Gait speed	Balance	5TSTS	30s CST	ABC	Icon-FES	LLFDI-FC
Spearman’s rho *p*-value								
SPPB	–							
Gait speed	**0.671** **< .001**	**–**						
Balance	**0.620** **< .001**	**0.436** **< .001**	**–**					
5TSTS	**−0.684** **< .001**	−0.148 0.242	−0.186 0.141	**–**				
30s CST	**0.623** **< .001**	**0.382** **0.002**	0.228 0.068	**−0.716** **< .001**	**–**			
ABC	**0.438** **< .001**	**0.337** **0.005**	**0.330** **0.006**	**−0.288** **0.021**	**0.335** **0.006**	**–**		
Icon-FES	**−0.519** **< .001**	**−0.527** **< .001**	**− 0.261** **0.033**	**0.285** **0.022**	**− 0.470** **< .001**	**−0.804** **< .001**	**–**	
LLFDI-FC	**0.512** **< .001**	**0.500** **< .001**	**0.349** **0.004**	**−0.269** **0.031**	**0.380** **0.002**	**0.745** **< .001**	**−0.787** **< .001**	–

Correlations that are statistically significant (*p* < 0.05) are indicated in bold

*SPPB* Short Physical Performance Battery, *5TSTS* Five times sit-to-stand, *30s CST* 30 s chair stand test, *ABC* Activities-Specific Balance Confidence Scale, *Icon-FES* Iconographical Falls Efficacy Scale, *LLFDI-FC* Late-Life Function and Disability Instrument-function component

Change scores, between pre- and post-assessment, showed no significant correlations between performance-based measurements and self-reported scales (Table 5). There were also no significant associations between self-rated perceived improvements in balance or leg strength and changes in gait speed, balance, leg strength, self-reported falls-efficacy and functional ability. However, the change in self-reported scales ABC, Icon-FES, and LLFDI-FC all correlated significantly with each other as did the performance-based measurements gait speed, balance, 5TSTS, and 30s CST with the total SPPB score.

**Table 5:** Correlations between change scores in performance-based measurement of gait speed, balance and functional leg strength with self-reported scales for falls-efficacy and functional ability, and self-rated balance and strength

	SPPB	Gait speed	Balance	5TSTS	30s CST	ABC	Icon-FES	LLFDI-FC	Self-rated balance	Self-rated leg strength
Spearman’s rho *p*-value										
SPPB	–									
Gait speed	**0.365** **0.011**	–								
Balance	**0.559** **< .001**	0.201 0.171	–							
5TSTS	**−0.703** **< .001**	−0.097 0.511	−0.115 0.435	–						
30s CST	**0.519** **< .001**	0.106 0.478	0.140 0.349	**−0.643** **< .001**	–					
ABC	0.219 0.135	−0.012 0.934	0.117 0.430	−0.069 0.641	0.037 0.806	–				
Icon-FES	−0.201 0.171	−0.181 0.218	− 0.167 0.257	0.035 0.814	0.104 0.487	**−0.479** **< .001**	**–**			
LLFDI-FC	0.031 0.834	−0.037 0.804	0.112 0.448	0.022 0.881	−0.110 0.461	**0.298** **0.030**	**−0.407** **0.002**	–		
Self-rated balance	0.084 0.571	−0.206 0.161	0.037 0.801	−0.095 0.520	0.257 0.081	0.024 0.862	0.023 0.870	−0.052 0.713	–	
Self-rated leg strength	0.106 0.472	−0.261 0.074	0.058 0.695	−0.129 0.383	0.250 0.090	0.001 0.996	0.052 0.712	−0.059 0.674	**0.693** **< .001**	–

Correlations that are statistically significant (*p* < 0.05) are indicated in bold

*SPPB* Short Physical Performance Battery, *5TSTS* Five times sit-to-stand, *30s CST* 30 s chair stand test, *ABC* Activities-Specific Balance Confidence Scale, *Icon-FES* Iconographical Falls Efficacy Scale; *LLFDI-FC* Late-Life Function and Disability Instrument-function component

### Measured effects of the intervention

Outcome measures at pre- and post-assessments for the whole sample and per group are presented in Table 2. Functional leg strength was significantly improved with substantial effect sizes (5TSTS *r*_rb_ = − 0.65, 30s CST *r*_rb_ = 0.76) following four months of either self-managed exercise intervention. The effect size for total SPPB score was smaller (*r*_rb_ = 0.34). Small effect sizes were seen for gait speed, balance, fall-efficacy and self-reported functional ability (*r*_rb_ < 0.21). Participants in both groups self-reported improved balance (in median 4, on a scale of 1–5) post intervention. Participants in the DP group were significantly more inclined than participants in the PB to agree that their leg strength had increased. No other significant differences between the two programmes were found.

For the whole sample, improvements in functional leg strength correlated significantly with self-reported exercise time, 30s CST (rho 0.393; *p* = 0.006) and 5TSTS (rho − 0.415; *p* = 0.003). Although total SPPB did not improve significantly, the outcome was correlated to reported exercise time (rho 0.462; *p* < .001). No significant associations were seen between exercise time and outcomes for gait speed (rho − 0.063), balance (rho 0.159), ABC (rho 0.078), Icon-FES (rho − 0.079) LLFDI-FC (rho 0.04), or with self-rated improvements in leg strength (rho 0.202) or balance (rho 0.264).

## Discussion

In this sample of independent high functioning community dwelling older adults there was limited room to measure improvement, in the performance-based balance test within the SPPB, self-reported balance confidence (ABC) or fear of falling (Icon-FES). In these tests, a large proportion of the participants obtained either close to the best or the best scores at pre-assessment. These ceiling effects were not seen in the self-reported functional ability according to LLFDI-FC. The measurements of functional leg strength according to time for 5TSTS or number of 30s CST, have in themselves no maximum score, and are thus not subject to ceiling effects. Using these outcome measures, substantial effect sizes were seen for functional leg strength, which also correlated with exercise time (minutes accomplished). Associations between performance-based measurements and self-report scales were seen at pre-assessment but not for change over time.

The ABC scale demonstrated ceiling effects, which is in accordance with previous studies [46–48]. Myers et al. [46] have shown that ABC scores above 80 are indicative of high functioning, usually physically active older adults who are unlikely to show further improvement in balance confidence. Ceiling effects were also found in Icon-FES. This scale includes a wider range of activities and situations than the original Falls Efficacy Scale (FES) [49], as well as pictures to clarify the context of the activity [32]. A score of 30–40 on the 30-item Icon-FES has been suggested to demonstrate a low concern of falling [50]. The large proportion of participants in our study within the categories of high balance confidence in the ABC scale and low concerns regarding falls in the Icon-FES indicates that these scales might not be suitable for detecting changes in relatively high functioning older adults as in our study, and the target population for fully self-managed falls prevention interventions. Ceiling effects have not previously been demonstrated for Icon-FES [32, 50, 51]. This scale was translated into Swedish for use in our study and included some linguistic adaptations, so there may be a further need for evaluation of the psychometric properties of this Swedish version. A short intervention period can also have an impact on the limited effect on fear of falling, according to a previous meta-analysis [52]. LLFDI-FC did not show a ceiling effect in our study, which is in line with results from previous studies [36, 53]. The instrument cover several functions on different levels which reduces the risk of reaching a ceiling effect.

The SPPB was used to measure lower-extremity function. The psychometric properties of this assessment battery have previously been extensively evaluated and SPPB is strongly recommended as the first choice of outcome measure in older community-dwelling persons [21]. The participants in our study increased their total score slightly and more than half of the participants showed an improvement larger than the previously published MCID [40]. Thus, our results support the use of SPPB when the purpose is to get a measurement of overall lower-extremity function. However, the balance sub-component showed strong ceiling effects, which is important to take in consideration. The sub-component gait speed intriguingly deteriorated in 29% of participants. This might be explained by that the intervention took part during winter months when conditions are challenging to take outdoor walks, or, that the programme focuses on strength and balance exercises and did not include taking walks. Some sub-components of the SPPB showed large effect sizes, in particular the 5TSTS. Hence the difference in total score hid an important improvement in an outcome measure with a strong predictive risk of falls. We also assessed functional leg strength and endurance using the 30s CST which confirmed the improvement. Almost half of the participants improved by at least 2 stands, which represents the MCID. Consequently, both of these tests are suitable to use in evaluations of intervention effectiveness, in groups similar to our. In contrast, 70% of the participants reached the maximum balance score of SPPB at pre-assessment, so there was an evident ceiling effect. This has previously been noted by others [12–14], and the test battery was modified to be more demanding in one study [14]. In fall-preventive interventions for community-dwelling older adults, in which balance exercise tasks are a major focus, objective balance measurements with higher sensitivity to change are needed. In the near future we may have reliable and valid measurement tools based on digital technology with built-in sensors in smartphones that are easy to use at a low cost [54]. Such sensor-tests may also provide opportunities for self-assessments [9–11], which could be used in self-management of exercise interventions.

In contrast to performance-based measurements of balance the self-rated change in balance, with questions made for this study [19], indicated improvements. This type of scale, which is used to rate perceived changes, like the Patient Global Impression of Change, has been criticized for participants’ recall bias [55]. Nevertheless, it allows the individual participant to express a change that is relevant or noticeable for them in their daily life, which may reflect a more real-life situation than a static balance test in the performance-based measurements. In addition, self-rated measures of change are sensitive to change and have been shown to correlate with changes in well-established instruments, at least concerning for example experiences of pain [56], quality of life [57] and physical function [15]. However, in our study the perceived change in balance and leg strength did not correlate with other measures of change. Most participants experienced that balance had improved while their perceived leg strength remained unchanged, which is in contrast to the performance-based assessments. This might be a result of that the performance-based balance assessment had a ceiling effect, and functional leg strength tests were sensitive to detect minor changes that the participant may not have noticed. To our knowledge, self-rated change in balance and strength has not previously been evaluated. Perhaps an improvement in balance is more easily noticed. Or maybe the two constructs, balance and muscle strength, are excessively intertwined to notice a difference between the two. To further develop and evaluate assessments of perceived change in balance and muscle strength more research is needed.

At pre-assessment significant associations were seen between performance-based measurements and self-reported scales. Previous cross-sectional studies have also shown associations between balance confidence (ABC) and self-reported function (LLFDI-FC), with various balance, physical performance measurements and self-rated health [38, 46]. However, looking at change scores, no correlations were found. This was not surprising given that both assessed balance and questionnaires for balance confidence and falls concern showed signs of ceiling effects, and thus small effect sizes in most outcomes which limits the conclusions that can be drawn from this correlation analysis. The exercise interventions were relatively short (4 months) and a small change in performance measures such as leg strength may not automatically and directly be reflected in falls efficacy or functional ability, as such process may take longer time [52]. There is a need for future longitudinal studies to further investigate associations between change scores in performance-based measurements and self-reported scales. However, changes in ABC, Icon-FES, and LLFDI-FC did correlate with each other, which supports the construct validity of these scales.

No differences were seen in the additional analyses between groups using the DP and the PB, except that DP participants were more inclined to agree that their leg strength improved post intervention. This is in line with our previous reported results showing that adherence to the programme were similar in both groups among participants who completed the intervention [19]. The results in the present study also showed that improvements in performance-based functional leg strength, the only significant improvement seen, were related to the amount of exercise, which implies a dose response relationship that could be further explored in a larger study.

### Methodological considerations

The majority of study participants were women (72%), a distribution normally seen in studies concerning falls prevention [2]. The inclusion and exclusion criteria allowed older adults with a broad range of characteristics to enrol in this study. We selected measurements and scales that have been used in falls prevention studies that included community-dwelling older adults, except for the self-rated perceived change in balance and strength questions made for this study. Despite the broad inclusion criteria in our study, ceiling effects were seen particularly in the performance-based assessments of balance and the self-reported balance confidence and falls concern scales, as most participants were high functioning. Despite the ceiling effects in these measures, more than 90% reported perceived decreased balance and almost 2/3 reported a fall the previous year, suggesting they are an important population to focus primary falls prevention. Consequently, there was limited room for evaluation of improvements, which needs to be considered in interpretation of effect sizes and for future evaluations of similar interventions. The results of this study put emphasis on the heterogeneity of older adults and that measurements to assess a wide range of physical function are needed. They also highlight the importance of measures that will be useful for evaluating self-managed interventions, as for example digital self-assessments and self-reported outcome measurements.

The use of MCID is based on previous studies where both length of the intervention and study population have to be considered. The reference values used in this study were obtained from studies that varied in length from nine weeks to one year follow-ups [40–43]. All MCID papers included a general older adult population, except for 30s CST where participants had hip osteoarthritis [42]. Thus, the results on proportion reaching a MCID in this study have to be interpreted with some caution although MCIDs are valuable to use as reference for important change.

A limitation with the present study was that it was a feasibility study with a relatively short intervention, and was not powered to evaluate exercise effects between the two programmes. Although, the four-month self-managed falls prevention exercise study showed some positive effects on self-reported balance and measured leg strength in both groups combined. We also created new questions on self-reported change in functional ability as no evaluations of such questions were found in the literature. The PB was used as a comparative group to the new DP and the aim was explore the feasibility of the outcome measures.

We did not apply any strict limits for what was considered a ceiling affect. Commonly ceiling effects are described when the majority of values obtained for a variable approach the upper limit of the scale. A criterion often applied is that ceiling effects are considered if more than 15% of respondents achieve the highest possible score [58]. Such guidelines are however difficult to apply in scales with high maximal scores, as in this study the ABC with a maximal score of 100%. In these situations, it is more complex to set definite limits for when the majority of values is considered to approach the upper limit of the scale. We decided to make these judgements based on the distribution of the scores described by median values and the third or first quartile in combination with results of previous studies indicating limits for meaningful effects [46, 50].

A strength of this study was that data was collected before and after the intervention by the same small group of assessors. Experienced physiotherapists assessed performance-based measurements blinded to results of self-rated scales and to group allocation. For ABC, individual verbal instruction was provided as the original scales have extensive written instructions. The participants answered all self-report questionnaires independently, with a researcher close by in case of any questions. The LLFDI-FC also had individual verbal instructions, yet many participants found the parted page for function unclear, which required some explanation. Several participants had questions regarding some illustrations in Icon-FES. For example: How concerned are you about crossing the street on a pedestrian crossing against the lights? How concerned are you when cleaning the gutter? These were all things that many participants were warned to do and never did. Consequently, they had objections to answering these questions, resulting in a few contradicting answers in this questionnaire.

## Conclusion

The outcome measures used to assess participants changes following two self-managed falls prevention interventions showed limitations. The balance sub-component of the SPPB and the self-reported measures ABC and Icon-FES might not be suitable for assessments in all community-dwelling older adults due to risk of ceiling effects. The LLFDI-FC did not show any ceiling effects, and could be used as a self-administered instrument. The 5TSTS and 30s CST were feasible in this study including high-functioning older adults. Both leg strength assessments were able to detect improvements and also showed correlation with exercise time, suggesting adequate level of challenge to reflect the capacity/function of relatively high-functioning older adults. Associations were seen at pre-assessment for performance-based and self-reported scales, considering change-scores no significant correlations were seen. More research is needed to identify suitable instruments for assessment of balance performance, and balance confidence in self-managed fall-preventive interventions in this group of older adults. Additionally, it is desirable to explore the participants’ own experiences of improvements in balance and strength in further studies.

## Supplementary Information

**Additional file 1: Supplementary Table (S1).** Template for Intervention Description and Replication (TIDieR) checklist for Safe Step feasibility study.

## Data Availability

The dataset used to support the findings of this study is not publicly available due to lack of consent for sharing individual data but are available from the corresponding author on reasonable request.

## Abbreviations

5TSTS: Five times sit to stand
30s CST: 30 s chair stand test
ABC: Activities-specific Balance Confidence scale
DP: Digital Programme
Icon-FES: Iconographical Falls Efficacy Scale
LLFDI-FC: Late Life Function and Disability Instrument - function component
MCID: Minimal Clinically Important Difference
PB: Paper Booklet
SPPB: Short Physical Performance Battery
